# Dental age assessment: A proposal for refining and extending the Demirjian stages for third molar mineralization evaluation

**DOI:** 10.1007/s00414-026-03819-9

**Published:** 2026-04-30

**Authors:** Maximilian Timme, Andreas Olze, Julian Wirtz, Sven Schmidt, Andreas Schmeling

**Affiliations:** 1 Institute of Legal Medicine, University Hospital Muenster, Röntgenstraße 23, 48149 Münster, Germany; 2 Institute of Legal Medicine, Charité-Universitätsmedizin, Turmstraße 21, 10559 Berlin, Germany

**Keywords:** Age estimation, Dental age, Orthopantomogram, Mincer, CBCT

## Abstract

Dental methods are now an integral component of forensic age assessment procedures. The stage classification for evaluating the mineralization of third molars in radiographs, as originally proposed by Demirjian et al. (1973), is well-established for this purpose. In practice, a modified version of this classification, based on the work of Mincer et al. (1993), is commonly employed. However, the verbal definitions of the stages exhibit notable inconsistencies. This method paper introduces a revised stage classification. Specifically, the stage definitions have been clarified, and the guidelines for applying the classification have been updated to align with the digital age. Additionally, new schematic illustrations are provided. These modifications aim to improve the reliability of the classification's application. In a second approach, four new intermediate stages—some of which have been previously proposed by other researchers—have been incorporated into the classification. The inclusion of these additional stages seeks to enhance the accuracy of age estimation. Whether the modified stages and the newly introduced intermediate stages provide significant added value will need to be determined through future studies.

## Introduction

Age assessment represents a current research focus within the forensic sciences [1]. Forensic age assessment is attracting growing attention because there has been a rise in the number of expert assignments due to increasing migration movements [2, 3]. It can be assumed that age assessment will continue to be of great relevance in the future.

For age assessments of adolescents or young adults, the Study Group on Forensic Age Diagnostics (AGFAD) proposes a combination of methods in the sense of a three-step procedure [4, 5]. Part of this combination of methods should always be a dental examination with determination of the dental status and X-ray examination of the dentition. The main criterion in the named examination of dentition is the assessment of the mineralization of third molars [4, 6].

Various classifications have been published to assess the mineralization of third molars [7–17]. Although the classifications all reflect tooth mineralization in a purely descriptive manner, they differ from each other to a certain extent significantly. The stage classifications differ, for example, in the number of stages, the definition of each stage, and the presentation. In addition, some methods require the determination of distances or ratios, while other methods do not need measurements.

A comparison of the 5 most common staging classifications by Olze et al. in 2005 revealed that the 1973 Demirjian et al. classification is most appropriate for forensic age assessment because it performed best not only for observer agreement but also for the correlation between estimated and true age [12, 18]. Olze et al. (2005) argued that this is due to the fact that the Demirjian classification is based on a sufficient number of stages which are defined independently of speculative estimations of length. In conclusion, this led to the recommendation that the classification of Demirjian et al. (1973) should be used to assess the mineralization of third molars in the context of forensic age assessment procedures [18]. Moreover, the Demirjian staging system was applied to second molars, for instance to support age assessment in specific age groups [19, 20].

The 8 individual Demirjian stages (A-H) for assessing the mineralization of molars originate from a paper published in 1973 [12]. The Demirjian stage classification discussed here should not be confused with the Demirjian *method* [12]. In the Demirjian *method*, teeth from the first incisor to the second molar of the left mandible are evaluated and assigned a stage from A-H. Thus, multiple teeth are always considered for each age assessment, explicitly not considering the third molar. Depending on the tooth, a score value is determined for each stage in turn. The score values of the individual teeth are then added together. This sum results in the so-called “maturity score”. Each maturity score is then assigned an age in years with one decimal place, separately for girls and boys.

It must be taken into account that the Demirjian *method* focuses on a younger age range between 3.0 and 16.0 years [12]. Overall, however, the method published by Demirjian et al. in 1973 already basically comprises the 8 stages A-H and the rules for their application.

The definition of one individual stage was already adjusted for the first time in 1980 by Levesque and Demirjian [21]. The authors redefined stage E in this context. While the 1973 description of stage E stated that the root length should be less than the crown height, the 1980 definition specified that the root length should attain a minimum of one third of the crown height. Remarkably, Demirjian himself used the old stage E in a 1986 book chapter without mentioning the updated stage definition [22].

The application of the stages from the Demirjian method to third molars goes back to Levesque et al. (1981) [23]. In 1981 the authors, who also include Demirjian himself, refer to the Demirjian method from 1973 [23].

In 1993, Mincer et al. applied a modified version of the Demirjian classification to third molars (Table 1) [24]. In the modification, the schematic representation seems to be very similar to Demirjian's from 1973. In contrast, the written definitions differ in part considerably. Moreover, since the rules are not mentioned in their paper, it remains unclear whether Mincer et al. adopted the additional stage determination rules proposed by Demirjian et al. (Table 2).

**Table 1.**
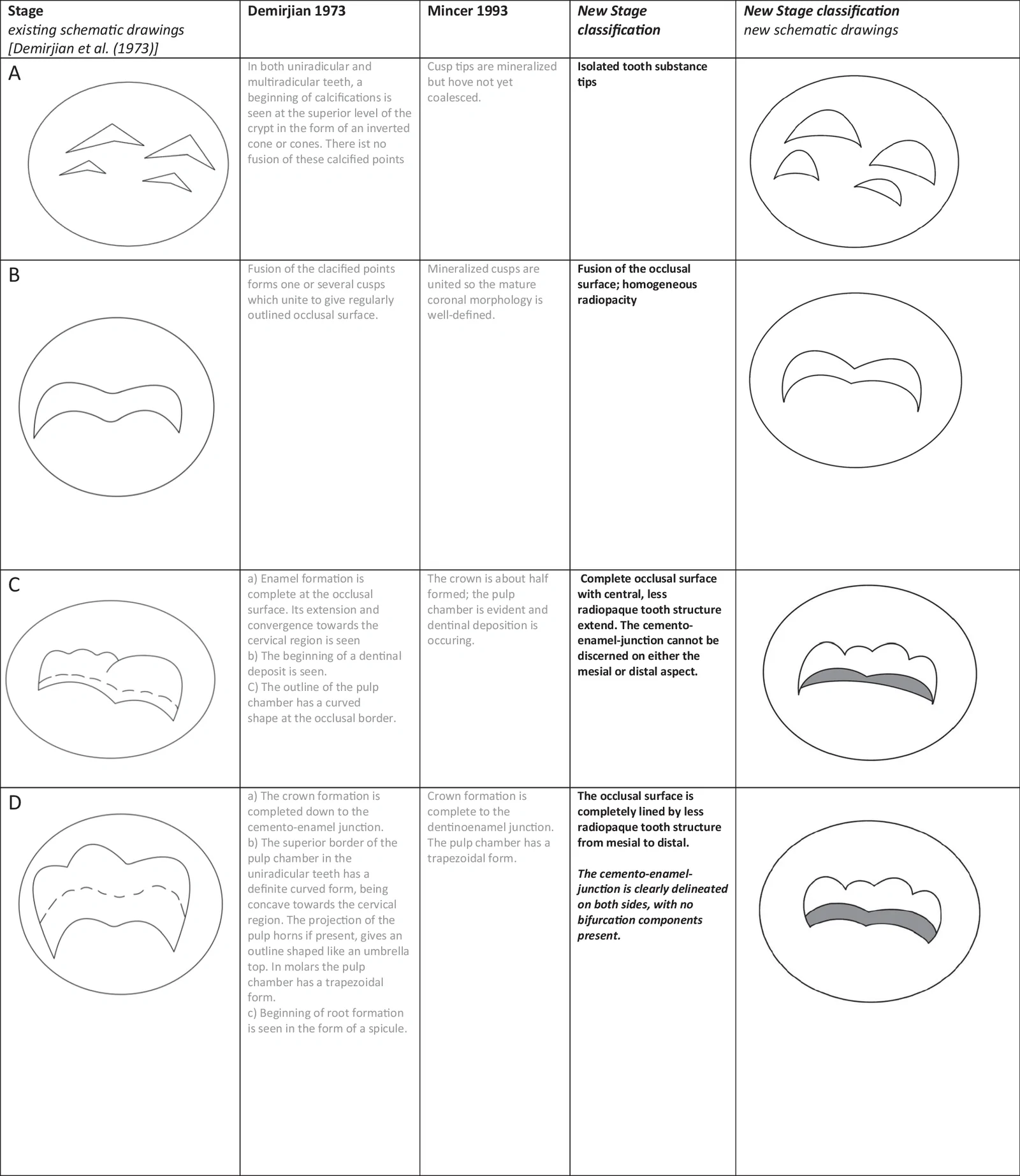

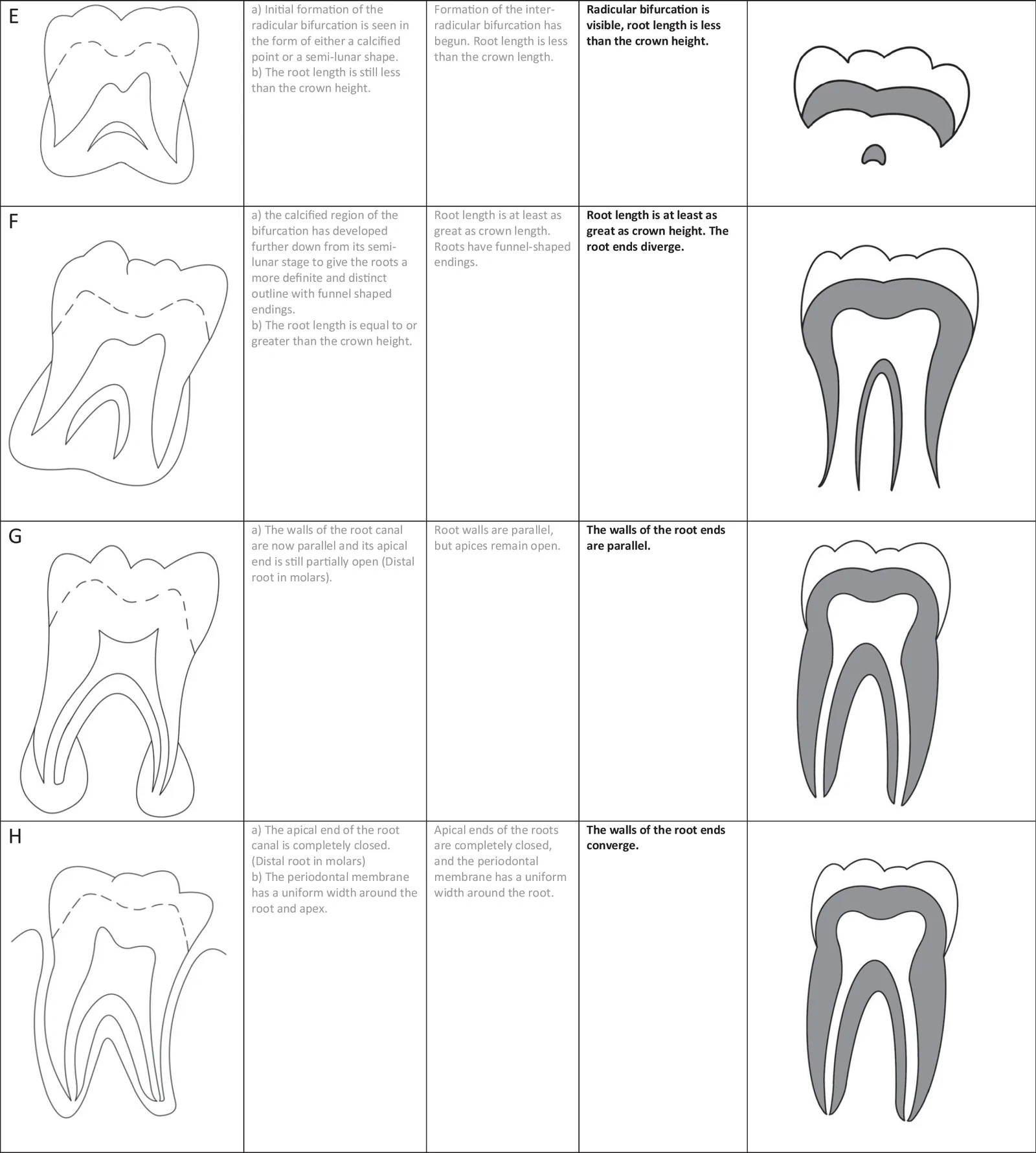
Schematic drawings and definitions of the stages of crown and root formation to score third molar mineralization. Direct comparison of the stages of Demirjian et al. (1973), Mincer et al. (1993), and the proposed new stages. Existing schematic drawings from Demirjian et al. 1973

**Table 2:** Rules canon for assessment the third molar mineralization according to Demirjian et al. 1973 and the new rules in comparison

**Rules for assessment according to Demirjian et al. 1973** (selection with relevance for the third molars)	**New rules for assessment**
-All teeth are rated on a scale A to H. The rating is assigned by following carefully the written criteria for each stage, and by comparing the tooth with the diagrams and Xray pictures given. The illustrations should only be used as an aid, not as the sole source of comparison. For each stage there are one, two or three written criteria marked a), b), c). If only one criterion is given, this must be met for the stage to be taken as reached; if two criteria are given, then it is sufficient if the first one of them is met for the stage to be recorded as reached; if three criteria are given, the first two of them must be met for the stage to be considered reached. At each stage, in addition to the criteria for that stage, the criteria for the previous stage must be satisfied. In borderline cases the earlier stage is always assigned -There are no absolute measurements to be taken. A pair of dividers is sufficient to compare the relative length (crown/root). To determine apex closure stages no magnifying glass is necessary. The ratings should be made with the naked eye -The crown height is defined as being the maximum distance between the highest tip of the cusps and the cemento-enamel junction. When the buccal and lingual cusps are not at the same level, the midpoint between them is considered as the highest point	-The image section may be enlarged for evaluation -The image contrast may be adjusted for evaluation -Automatic soft- and hardware filter- or focus-systems (depending on the manufacturer of the X-ray device) may be used—but must be specified, especially in the context of studies -In borderline cases between two possible stages, the lower one must be chosen -Teeth tilted in the lingual-vestibular direction cannot be assessed if they compromise stage classification -In the case of angled teeth, the tooth axis—starting from the complete occlusal surface—must be taken into account -Pictograms are only considered as a supplement; the written definition is decisive -There are no absolute measurements to be taken -The crown height is defined as being the maximum distance between the highest tip of the cusps and the cemento-enamel junction. When the buccal and lingual cusps are not at the same level, the midpoint between them is considered as the highest point -The root length is determined between the cemento-enamel junction and the most apically detectable hard tooth substance -In case of differences in the root development, the more developed root is decisive for the evaluation

To allow for direct comparison, Table 1 juxtaposes the stages defined by Demirjian et al. and those proposed by Mincer et al. [12, 24].

Upon detailed examination of the classifications, the following differences can be highlighted:

- Assessment of the distal root only (Demirjian)
- Evaluation without magnifying glass with the naked eye (Demirjian)
- Illustrations only as additions; the text definition is leading (Demirjian)
- Several criteria within the stages with different weighting (Demirjian)
- In borderline cases the earlier stage is always assigned (Demirjian)
- Stage B: coronal morphology (Mincer) vs. occlusal surface (Demirjian)
- Stage C: the crown is about half formed (Mincer) vs. enamel formation is complete at occlusal surface with extension and convergence towards cervical region (Demirjian)
- Stage D: dentinoenamel junction (Mincer) vs. cementoenamel junction (Demirjian)

Another modification of the stages of Demirjian et al. was published by Tompkins in 1996 [25]. Tompkins arrives at a total of 13 (sub-) stages through further subdivisions [25]. Tompkins evaluates the absence of a calcifying tooth germ as a separate stage. Beyond that, Tompkins emphasizes that the modifications were necessary in order to be able to evaluate taurodontic teeth [25]. However, it must be taken into account that the influence of taurodontism on tooth development has not been conclusively clarified [26]. This is therefore to be viewed critically, since taurodontism can be based on genetic changes that may influence tooth development in general [27, 28]. Consequently, it is a questionable issue whether taurodontic teeth should be used for age assessment at all. The question is of great interest because the prevalence of taurodontism varies widely around the world [29–31]. For example, prevalences of about 2 percent have been described for Germany [32], while in Senegal, for example, up to 48 percent have been reported [33]. For a Chinese cohort, a prevalence of 46 percent was found [31, 34]. In view of these partly high prevalences of taurodontism, it becomes clear that clarification of a possible influence on tooth development is urgently needed [26].

In 2002, another modification of the Demirjian stages was presented by Solari and Abramovitch [35]. In concrete terms, it is rather a further development of the Mincer stages [24], which were essentially adopted by Solari and Abramovitch [35]. The modification by Solari and Abramovitch consists in the extension of the Mincer classification by the two "borderline" stages F_1_ and G_1_. The authors intended the introduction of the new stages to provide a more accurate age estimate around the age of 18 years [35].

- F_1_: Root length is at least twice crown length. The roots still have a funnel-shaped ending
- G_1_: Root walls are parallel, but apices are not entirely closed. The periodontal ligament space at the apical ending is ≥ 1.0mm

A classification for the mineralization of third molars which is unambiguous for all users is elementary for practical use in forensic age estimation. Since the authors of the present article consider this to be true only to a limited extent for the Demirjian stages as well as their later modifications, a new adjustment of the stages and their rules for application is suggested.

### Aims

The aim of the work was primarily to develop an as unambiguous stage classification as possible, which can be applied without doubts by examiners who are independent of each other.

In addition, the rules canon for the application of the classification was to be updated and thus brought into line with contemporary requirements of the digital era.

Furthermore, additional stages should be proposed in order to increase the accuracy of the age diagnosis.

### Methodological proposal

Table 1 presents the new modification of the known Demirjian-Stages A-H.

For a better overview of differences, the new classification in Table 1 is directly contrasted with the classifications of Demirjian et al. (1973) [12] and Mincer et al. (1993) [24]. In contrast to Demirjian et al., sub criteria for the definition of stages are not provided. Furthermore, modified schematic drawings are presented. It is important that these drawings are not overrated in practical application. As in Demirjian et al. (1973) [12], they serve only for orientation. Also in the new classification, the written definitions of the stages are decisive.

Table 2 contains the new rules canon for the application of the classification. For clarification of the new rules, the rules in Table 2 are directly contrasted with the old rules according to Demirjian et al. from 1973 [12].

Table 3 presents the 4 new intermediate stages C1, E1, F1 and G1. For some stages modifications of the main stages were necessary. The modified main stages are marked with an asterisk. The intermediate stages follow their main stages in the following order: A – B – C – C1 – D – E* – E1 – F* – F1 – G – G1 – H*.

**Table 3.**
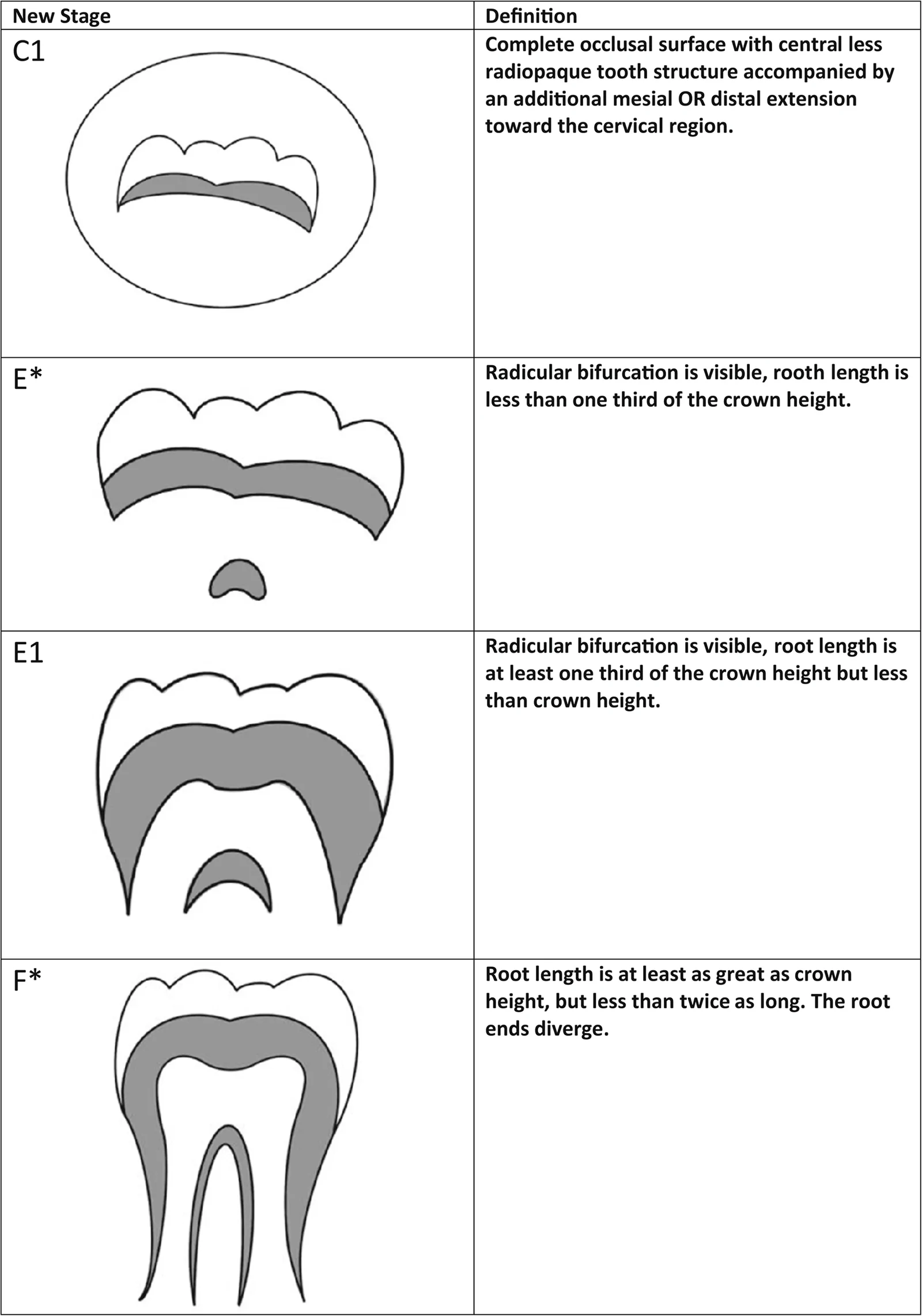

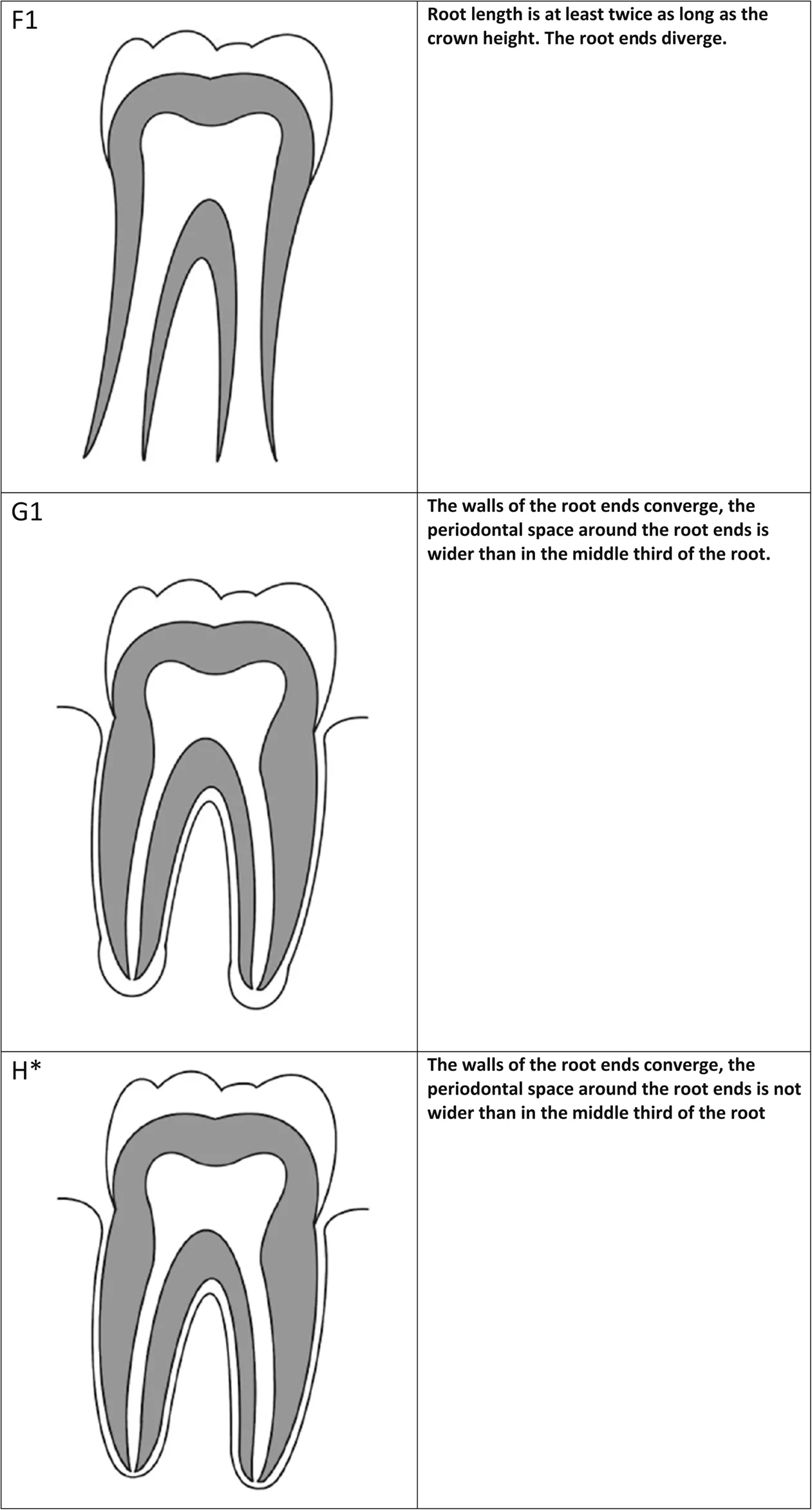
New intermediate stages for assessing the mineralization of third molars and correspondingly adjusted main stages, which are marked with an asterisk

## Discussion

### Modification of the existing stages

To the authors’ mind the Demirjian stages and their previous modifications show some shortcomings which are related particularly to the unambiguity of the individual stages.

One problem with the Demirjian classification is that reference was made to the shape of the pulp cavity or radicular bifurcation for several stages [12]. The interpretation of these shape descriptions has proven to be problematic in practice. However, it should be noted that upper third molars may have a trifurcation instead of a bifurcation.

Furthermore, it is questionable that complete closure of the apical foramen is required for the detection of stage H in Demirjian-classification. Complete closure of the foramen should not be demanded, since vessels and nerves enter the tooth apically throughout life [36]. Thus, stage H according to Demirjian et al. describes a state that, cannot be achieved in a vital tooth. It must be mentioned, however, that radiographic evidence of the foramen may indeed be impossible because of the very fine structure, yet its presence is anatomically certain [37]. Thus, in stage H, according to Demirjian et al., an incidental radiologic finding, usually produced by superimposition, becomes the criterion. Therefore, the 1973 wording is unfortunate and can lead to confusion.

Another issue is that Demirjian requires for stage H that the periodontal membrane has an equal width around the entire tooth. It must be taken into account that delineating the periodontal membrane on the orthopantomogram (OPG) often presents a challenge. However, in the staging system proposed in the present study, assessment of the periodontal membrane is not required, as the differentiation between stages F, G, and H is based solely on the configuration of the root apices. Whether the inclusion of the apical periodontal space determination will ultimately prove useful for the newly proposed stage G1 remains to be determined.

Since the formulation of the rules of Demirjian et al. can also lead to misunderstandings, a revision of these was also necessary. For example, Dermirjian et al. make no statements in their rules about dealing with tilted teeth. This gives the idea of a problem-free handling of such teeth. However, it must be taken into account that tilting changes the length conditions, as they are decisive in stages E and F, for example. Therefore, according to the new rules, tilted teeth should be excluded from the examination if they compromise stage classification (Table 2).

One rule of Demirjian et al. for the application of their stages stated that the distal root is decisive for evaluation. Based on the experience of the authors of the present article, it must be mentioned that this root is not necessarily the most developed one in practice. Therefore, future studies should examine whether the rule should be changed to consider the most developed root as decisive. Alternatively, an approach in which both roots are examined separately from each other is also feasible. Until this question is finally clarified, the authors have proposed to evaluate the most developed root (Table 2).

### Additional stages

With the new intermediate stages presented in Table 3, the new modification comprises a total of 12 stages. By this further differentiation, the authors expect a higher accuracy in age assessment in the sense of an improved correlation between stage and age. Solari and Abramovitch had already introduced "borderline" stages from this intention [35]. For the stages introduced by Solari and Abramovitch, it is considered problematic that a concrete distance is introduced as a criterion for stage G_1_, although the ability to manage without absolute length measurements is seen as a strength of the original Demirjian stages in the past [18]. In light of this consideration, the revised definition of stage G1 presented herein deliberately omits any reliance on specific quantitative measurements (Table 3).

In addition, the requirement that the root length be twice the crown length (requirement for stage F1 [35]) is, in the experience of the authors of this article, a condition that is basically seldom achieved in practice. This means that, by definition, the stage F1 does not necessarily always have to be reached. In fact, it is more likely that the stage will not be passed through as standard. The other intermediate stages from Table 3 should also be closely monitored in this regard in the future.

In contrast to the 1996 modification by Tompkins et al., the current extension of the classification does not introduce a stage that evaluates the absence of a calcifying tooth germ [25]. This must be viewed critically with Tompkins et al. modification, as third molars are often not formed or extracted, and their absence therefore does not correlate with age.

On the other hand, the increase in the number of stages by the new definition of intermediate stages is also problematic, since Olze et al. found in 2005 that the relatively low number of stages in Demirjian et al. compared to other classifications is a particular strength of the method [18]. Therefore, in the future, special attention should be paid to whether the increase in the number of stages actually brings added value in terms of better correlation with age.

### Modified schematic drawing

The existing schematic drawings, as presented by Demirjian et al. in 1973, have undergone minimal modifications over time when cited, likely driven more by copyright concerns than substantive reasons.

The newly created schematic drawings, as found in Tables 1 and 3, exhibit a stronger alignment with the radiomorphology of the findings compared to the older, pre-existing schematic drawings, which are also included for comparison in Table 1. Details in the drawings were further refined and aligned even more closely with the written definitions of the stages. The periodontal space was omitted at least for the new main stages, as it holds no relevance. Furthermore, ambiguities were resolved, such as the lack of clarity regarding why certain lines were depicted as dashed in the old schematic drawings. Instead, dentin and cementum were rendered in grey. In practice, however, it remains problematic that cementum and dentin cannot be reliably differentiated in X-ray images due to their comparable radiopacity, but only based on topographical criteria.

Our group assumes that the new drawings could potentially contribute to simplifying the application of the method in the future.

### Impact of the imaging technique

Since staging in practice is essentially applied on the OPG and most reference studies on the Demirjian-stages are also available for this X-ray technique, the impact of this imaging technique on the evaluability of the stages has to be discussed.

The OPG is a summation image in which all objects in the beam path are imaged superimposed [38, 39]. Due to the imaging technique, only a certain slice thickness can be imaged. This is approx. 20–25 mm in the molar region [38]. Objects outside this slice are increasingly blurred or not visible at all. In practice, this often applies to the apical tooth region, especially if the tooth is tilted in the vestibular-lingual direction or the roots kink towards the apex. These cases are regularly not evaluable in the classic OPG in terms of age assessment (Table 2).

In addition, the OPG is basically very susceptible to positioning errors of the examined person in the X-ray unit [38]. Regarding the stages in question, positioning errors, in the sense of tilting in combination with turning the person’s head in the X-ray unit, can lead to changes in the length ratios of the crown to the root. Errors of this kind would possibly lead to incorrect results when applying the stages. A good method for checking possible errors of this kind has proven to be controlling that the condyles are equal in height and the rami are equal in width [40].

Technological progress and digitization not only affect the evaluation of images, but also the generation of digital X-ray images. Some manufacturers equip their X-ray units with hardware or software tools that can significantly improve image quality. An example of a hardware solution is the Direct Conversion Sensor (DCS) from Dentsply Sirona (Dentsply Sirona Inc, Charlotte, North Carolina, USA). As an example of software solutions, Adaptive Focal Point (AFP) and Adaptive Gray Scale (AGS) from the company Morita (J. MORITA EUROPE GMBH, Dietzenbach, Germany) should be mentioned. These technologies can significantly improve the sharpness of detail [41]. Although not yet systematically investigated, it can be assumed that these modern technologies have an impact on the evaluation of dental panoramic radiographs in age assessment. Experience has shown that the demarcation of the periodontal membrane or the apical foramen using these technologies is significantly better. In the view of the authors, however, the use of appropriate technology, especially in the context of studies, should be clearly highlighted.

In some X-ray devices for OPG, the sharp layer can be selected (e.g. Orthophos Sharp Layer (SL) 2D; Dentsply Sirona Inc, Charlotte, North Carolina, USA). This allows initial blurred structures, if any, to still be seen in focus. However, this is not directly comparable to the standard OPG. Thus, these technologies are somewhat compatible with cross-sectional imaging techniques. It can be assumed that although the evaluability is significantly improved by these technologies, the results in staging also deviate significantly from classic OPGs.

OPGs in which an evaluation was only possible by manually shifting the layer are therefore more likely to be seen as cross-sectional images, which is crucial for the choice of reference studies. This applies equally to a corresponding technical development in which the sharp layer is automatically placed on the tooth structures by the device, in the sense of an autofocus function (e.g. Orthophos S 2D; 2D-CsI-Plus sensor with autofocus; Dentsply Sirona Inc, Charlotte, North Carolina, USA).

Overall, the OPG is a very special imaging technique, which shows clear limitations and is prone to artifacts. The examiner must be fully aware of the peculiarities of the OPG. It is important to emphasize that this procedure is nevertheless the basis for assessing the development of the third molars as advantages such as availability, low radiation exposure, assessability of the entire dentition and duration of the examination usually dominate. Thus, the OPG should still be considered the gold standard for dental age assessment.

The classifications for third molar mineralization mentioned in this article cannot be easily transferred to other imaging techniques. In any case, appropriate reference studies would be necessary here.

### Using Demirjian stage classification by means of 3D imaging

Studies using the Demirjian stages of third molars with three-dimensional imaging are available [42–46]. In particular, the cone-beam computed tomography (CBCT) should be mentioned here, which is becoming more and more widespread in the dental field.

The advantage of CBCT is that the layer with the structures to be examined can be selected freely. For example, the tooth apex can be precisely adjusted and evaluated, or the bifurcation or trifurcation in the maxilla can be focused on directly. One strength of CBCT could also lie in the fact that a higher rate of evaluability of the upper jaw third molars could be achieved. This is often difficult with OPG today due to superimposition effects.

Furthermore, the CBCT is isometric, which means that there are no size distortions. Since the volumes can be freely moved in space, positioning errors can also be easily compensated for. Whether these clear advantages are actually accompanied by a more accurate age prediction in contrast to the OPG should be clarified in the future by direct comparative studies. This is therefore of great interest, since it must be assumed that, despite the significant reduction in radiation in modern CBCT devices in the past, the radiation exposure during CBCT is still at least about 3–6 times higher than with an OPG [47, 48]. However, radiation exposure associated with CBCT imaging is highly dependent on the specific device used, the selected field of view (FOV), and various technique-related factors. Notably, effective doses up to 42-fold higher than those of comparable OPGs have been documented in the literature [49]. This aspect must be taken into account in relation to radiation protection.

Magnetic resonance imaging (MRI) can also be used to assess the Demirjian stages [50–53]. The great advantage of this technique is that it does not involve ionizing radiation. It is particularly noteworthy that development for dental-dedicated MRI (ddMRI) is being pushed forward and that the first ddMRI has been commercially available for some time (MAGNETOM Free.Max Dental Edition; Dentsply Sirona Inc, Charlotte, North Carolina, USA). However, the technique is much less widespread, very complex and, not least, very expensive. The overriding question is whether this effort is actually associated with an increase in the accuracy of age prediction compared to the OPG.

Overall, it must be clearly stated that three-dimensional imaging techniques for the assessment of Demirjian stages must never be employed on the basis of reference data collected for OPG imaging. It has already been shown that there are significant differences depending on whether 2D or 3D imaging is applied to determine the Demirjian stages [50, 54]. In contrast, another study could not demonstrate any difference in the stage assignment between OPG, CBCT and extracted tooth for the method according to Gleiser et al. (1955) modified by Köhler et al. (1994) [55]. In the case of using Demirjian stage classification, or modifications of it, imaging-appropriate reference data must be utilized.

## Conclusion

The present work highlights that even well-established approaches in age assessment remain amenable to refinement and underscores the need for continued, in-depth investigation in this field. The proposed modification of the Demirjian stage classification appears to enable a more precise allocation of individual developmental stages, thereby reducing ambiguities inherent in the original system. As a consequence, an improvement in the reliability of the method may be anticipated. Moreover, the introduction of clearly defined application rules facilitates its use within contemporary, digitally based assessment frameworks.

Whether the modified stages and the newly introduced intermediate stages provide a measurable additional benefit must be determined by future, appropriately designed studies. Finally, given the high prevalence of taurodontism in certain regions of the world, the influence of this condition on dental development and third molar mineralization warrants urgent and systematic investigation.

## Data Availability

No primary datasets were generated or analyzed during the current study. This work is based on a synthesis of data from previously published studies, which are cited within the article.
