# Correction to: Dental age assessment: A proposal for refining and extending the Demirjian stages for third molar mineralization evaluation

**DOI:** 10.1007/s00414-026-03835-9

**Published:** 2026-05-14

**Authors:** Maximilian Timme, Andreas Olze, Julian Wirtz, Sven Schmidt, Andreas Schmeling

**Affiliations:** 1 Institute of Legal Medicine, University Hospital Muenster, Röntgenstraße 23, 48149 Münster, Germany; 2 Institute of Legal Medicine, Charité-Universitätsmedizin, Turmstraße 21, 10559 Berlin, Germany

**Correction to: International Journal of Legal Medicine**

10.1007/s00414-026-03819-9

In the original version of this manuscript, the corresponding parts of both Table 1 and Table 3 were not correctly reordered: the first part was presented as the continuation of the table instead of as the initial part.

The correct versions of Tables 1 and 3 are shown below.

Correct start of Table 1

**Figure:**
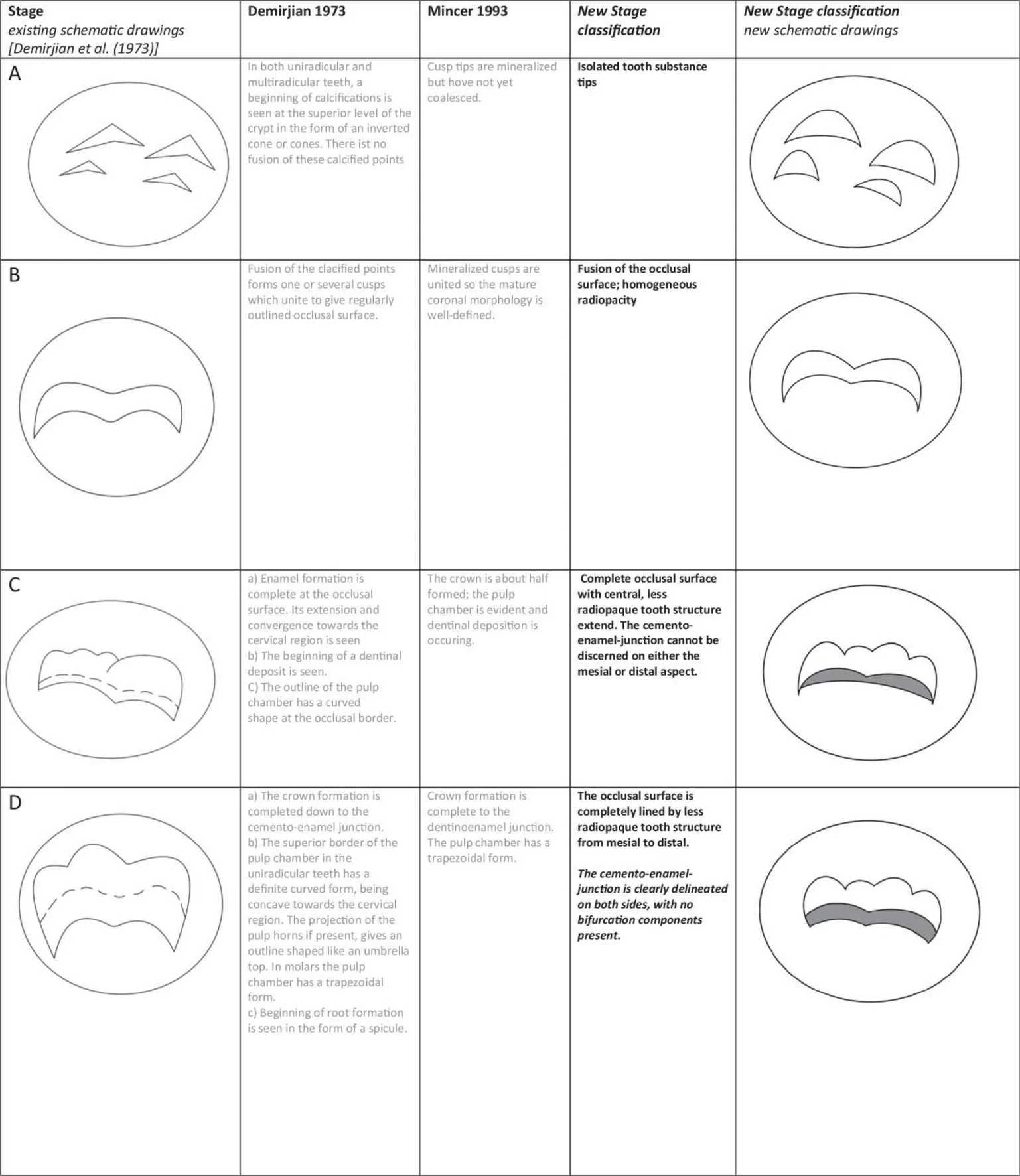

Incorrect start of Table 1- in fact the correct continuation -

**Figure:**
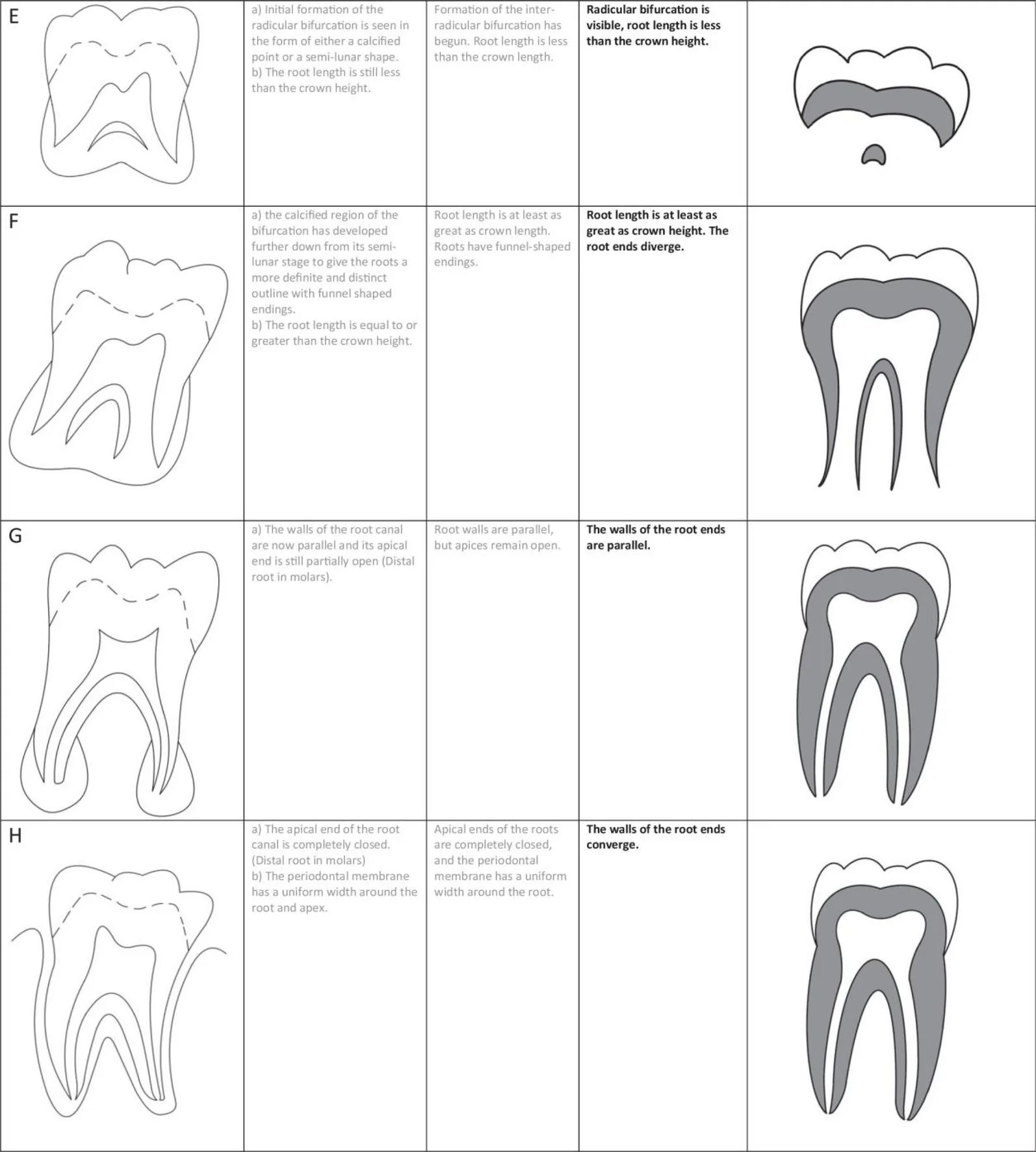

Correct start of Table 3

**Figure:**
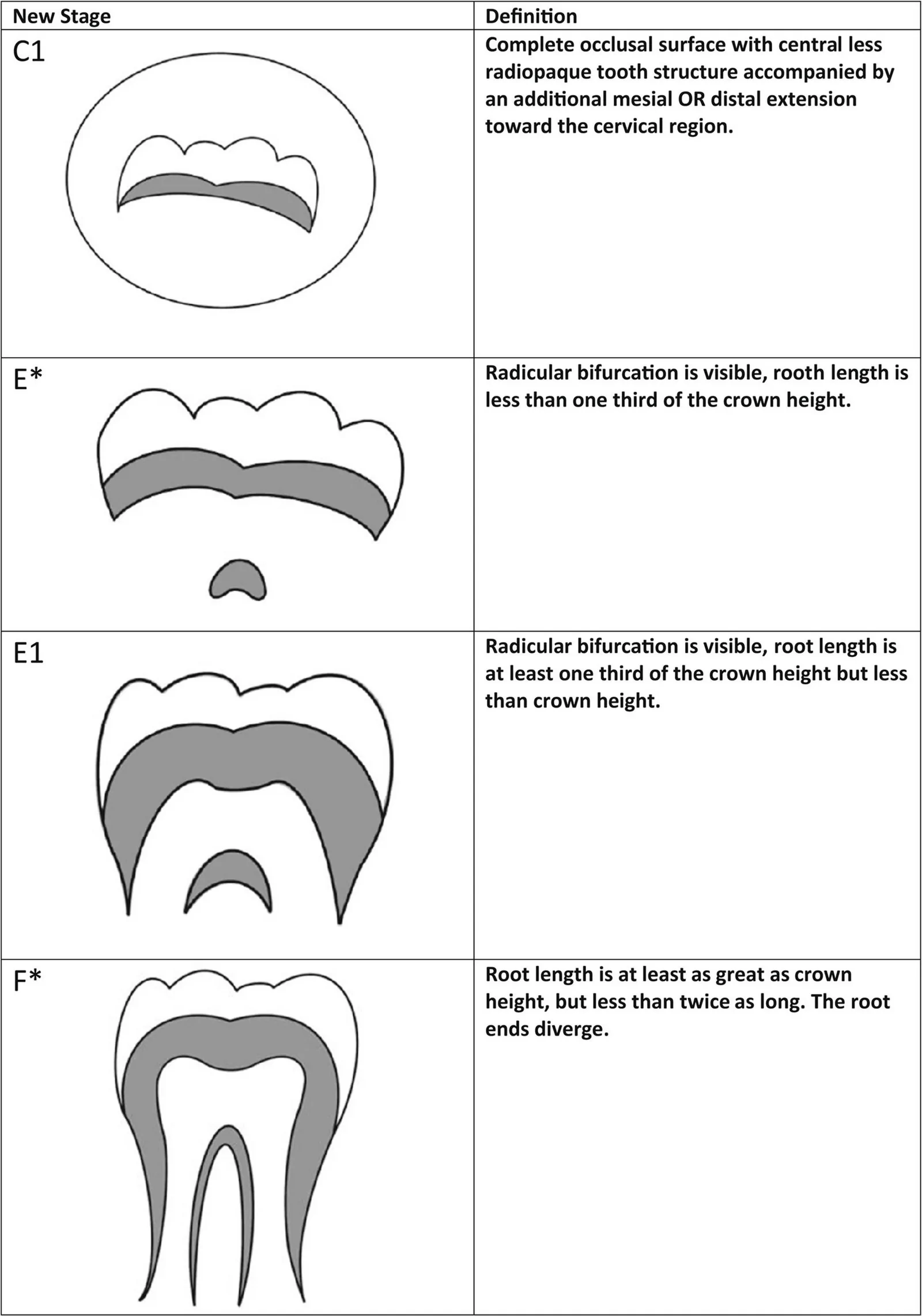

Incorrect start of Table 3 - in fact the correct continuation -

**Figure:**
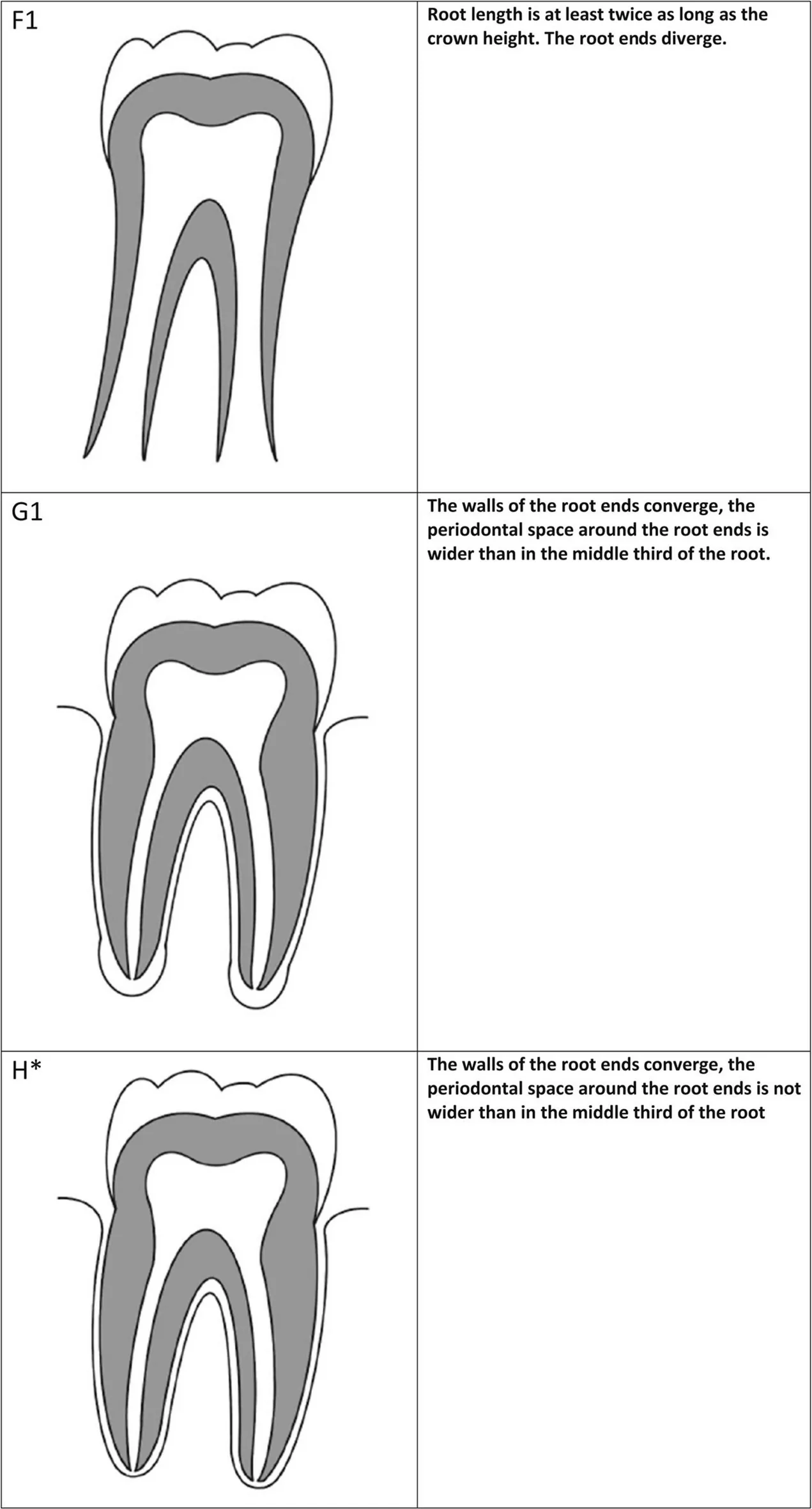

The original article has been corrected.

